# ﻿Revision of rove beetle genus *Bolitogyrus* Chevrolat (Staphylininae, Cyrtoquediini). Supplement 1

**DOI:** 10.3897/zookeys.1096.80773

**Published:** 2022-04-15

**Authors:** Adam J. Brunke

**Affiliations:** 1 Agriculture and Agri-Food Canada, Canadian National Collection of Insects, Arachnids and Nematodes, 960 Carling Avenue, Ottawa, Ontario, Canada Agriculture and Agri-Food Canada, Canadian National Collection of Insects, Arachnids and Nematodes Ottawa Canada

**Keywords:** Neotropical, new species, Oriental, Staphylinidae, taxonomy

## Abstract

*Bolitogyrus* is a moderately diverse genus of 78 species that are widely disjunct in the subtropical and tropical forests of the Neotropical and Oriental regions. Following recent revisions of both the Neotropical and Oriental species, this study provides new distributional data, a revised species concept for *Bolitogyrusstrigifrons* (Wendeler) **sensu nov.**, and the description of *B.pseudostrigifrons***sp. nov.** and *B.nigropolitoides***sp. nov.**, bringing the total number of *Bolitogyrus* species to 80. Several keys are updated to reflect the newly available data and new species.

## ﻿Introduction

*Bolitogyrus* Chevrolat (Staphylininae, Cyrtoquediini) is an uncommonly collected genus of predatory rove beetles. Its 78 extant species are specialists of humid microhabitats on and in fungusy deadwood within the forests of the Neotropical and Oriental regions (Brunke and Solodovnikov 2014; Brunke 2017). Twenty-eight species are distributed from Mexico to Ecuador (Brunke and Solodovnikov 2014), while the 50 Oriental species are distributed west of Wallace’s Line, from the Himalayan region and southwest India, east to Taiwan (Brunke 2017). Briefly widespread across the northern hemisphere during one of the short early Eocene hyperthermals, the range of *Bolitogyrus* shortly thereafter diverged into two lineages and further contracted southward to refugia as global climate polarized and temperate biomes emerged at higher latitudes during the Eocene–Oligocene transition (Brunke et al. 2017). Eocene fossils of the genus have revealed critical evidence of its past distribution in the Western Palaearctic (Baltic amber: *Bolitogyrusfragmentus* Brunke, Żyła & Solodovnikov) and North America (Green River Formation: undescribed taxon) (Brunke et al. 2017, 2019).

As a supplement to previous taxonomic revisions, the present work aims to publish new specimen data, refine concepts of described species, and describe two new species.

## ﻿Materials and methods

### ﻿Depositories

**cShi** Personal collection of Y. Shibata, deposited at the Museum of Nature and Science, Toshiba, Japan (S. Nomura);

**cSmet** Personal collection of A. Smetana, deposited at the Museum of Nature and Science, Toshiba, Japan (S. Nomura);

**CMN**Canadian Museum of Nature, Ottawa, Ontario, Canada (R. Anderson);

**CNC**Canadian National Collection of Insects, Arachnids and Nematodes, Ottawa, Ontario, Canada;

**IRSNB**Institut royal des Sciences Naturelles de Belgique, Brussels, Belgium (Y.Gérard, T. Struyve);

**NHMD** Natural History Museum of Denmark, University of Copenhagen, Denmark (A. Solodovnikov, J. Pedersen);

**NMPC**National Museum Prague, Prague, Czech Republic (J. Hájek);

**ROM**Royal Ontario Museum Collection, Toronto, Ontario, Canada (B. Hubley);

**SDEI**Senckenberg Deutsches Entomologisches Institut, Müncheberg, Germany (S. Blank);

**SEMC** Snow Entomological Collection, Biodiversity Institute, Kansas, USA (Z. Falin).

### ﻿Specimen data

Type label data are given verbatim, with labels separated by “/” and comments indicated in square brackets. Non-type label data were standardized to improve clarity. Specimens were georeferenced using Google Earth or Google Maps.

### ﻿Microscopy, illustration, and photography

All specimens were examined dry using a Nikon SMZ25 stereomicroscope. Genitalia and terminal segments of the abdomen were dissected and placed in glycerin filled vials, pinned with their respective specimens. Line illustrations were made from standard images and then digitally inked in Adobe Illustrator CC-2021. All imaging, including photomontage was accomplished using a motorized Nikon SMZ25 microscope and NIS Elements BR v. 4.5. Photos were post-processed in Adobe Photoshop CC-2021.

### ﻿Measurements and character variability

All measurements were made using a live measurement module within NIS Elements BR v. 4.5. Measurements were taken as listed below, but only proportional (HW/HL, PW/PL, EW/EL, PW/HW) and forebody measurements were stated directly in descriptions. Total body length is generally difficult to standardize for Staphylinidae and was not measured due to the contractile nature of the abdomen.

**HL** Head Length, at middle, from the anterior margin of frons to the nuchal ridge;

**HW** Head Width, the greatest width, including the eyes;

**PL** Pronotum Length, at middle;

**PW** Pronotum Width, greatest width;

**EL** Elytral Length, greatest length taken from level of the anterior most large, lateral macroseta to apex of elytra. EL approximates the length of the elytra not covered by the pronotum and therefore contributing to the forebody length;

**EW** Elytral Width, greatest width;

**ESut** Elytral Suture, apex of the scutellum to the apex of the elytra

**Forebody**HL + PL + EL.

## ﻿Taxonomy

### ﻿Staphylininae Latreille, 1802

**Cyrtoquediini Brunke & Solodovnikov, 2016**

#### ﻿ Bolitogyrus

Taxon classificationAnimaliaColeopteraStaphylinidae

Chevrolat, 1842

2C82244F-E4F4-5111-B0FF-ED01296EE25D

Bolitogyrus Chevrolat, 1842: 641. Type species Quediusbuphthalmus Erichson, 1840: 534, fixed by monotypy. Brunke and Solodovnikov 2014 (revision of Neotropical species); Cai et al. 2015 (Chinese species); Brunke 2017 (revision of Oriental species); Brunke et al. 2017 (biogeography, fossils); Brunke et al. 2019 (Baltic amber fossil); for extensive reference list, see Brunke and Solodovnikov 2014

### ﻿Neotropical species

**Buphthalmus group**

#### ﻿ Bolitogyrus costaricensis

Taxon classificationAnimaliaColeopteraStaphylinidae

(Wendeler, 1927)

7286683D-2806-5FC4-9C94-00F1AA39AD48

Cyrtothorax costaricensis Wendeler, 1927: 8
Cyrtothorax nevermanni Scheerpeltz, 1974: 181 (in key)
Bolitogyrus costaricensis (Wendeler): Brunke and Solodovnikov 2014 (redescription)

##### Non-type material.

**Nicaragua: Matagalpa Dept.**: 6 km N Matagalpa, Selva Negra, 12°9'54"N, 85°4'36"W, 1300 m, montane forest, beating, 19–22.V.2002, R. Anderson (5, CMN).

#### ﻿ Bolitogyrus erythrurus

Taxon classificationAnimaliaColeopteraStaphylinidae

(Kraatz, 1858)

1147CBE5-3559-5D32-AFEF-3A3EBA29AA4B

Fig. 1A–C

Cyrtothorax erythrurus Kraatz, 1858: 368
Bolitogyrus erythrurus (Kraatz): Brunke and Solodovnikov 2014 (redescription)

##### Type material.

***Syntype*** (1 female, SDEI): Nov. Gren. [green label script] / Coll. Kraatz [white label, printed] / erythrurus [green label, script] / Syntypus [red label, printed] / Syntype ♀, Cyrtothoraxerythrurus Kraatz, 1858, det A. Brunke 2013 [red printed label].

##### Non-type material.

Country unknown: “Nova Grenada” [handwritten label], erythrurus Kr. [handwritten label], “R. I. Sc. N. B.”, “17.479", “Coll. et det. A. Fauvel” (1 female, IRSNB).

##### Comments.

**Figure 1.**
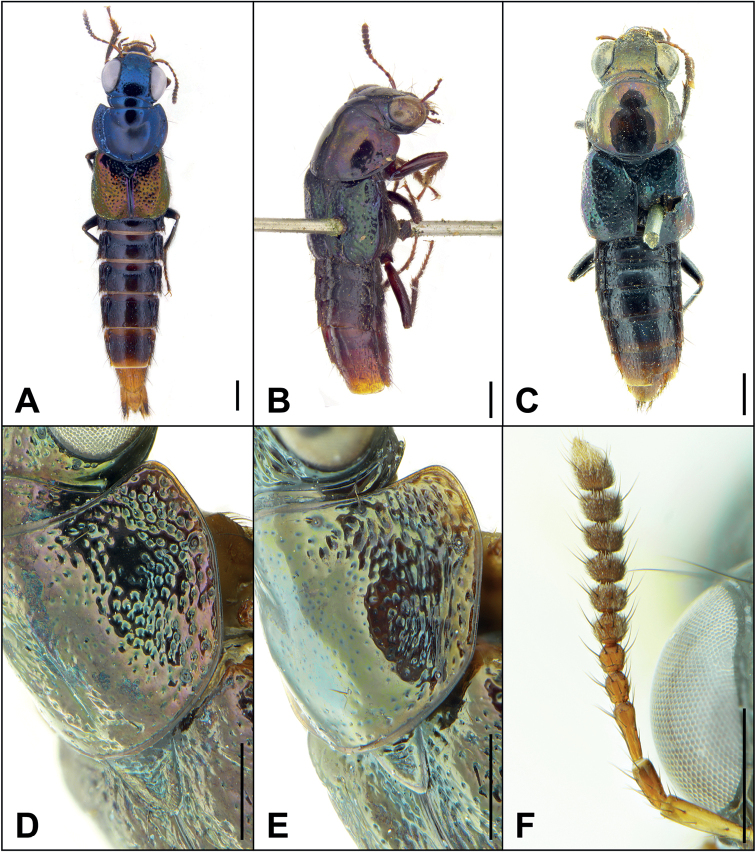
**A–C** habitus of *Bolitogyruserythrurus* (Kraatz) **A** non-type, Panama **B** syntype, “Nova Grenada” **C** non-type, “Nova Grenada” **D, E** pronotum, lateral view **D***B.viridescens* Brunke **E***B.pseudostrigifrons* Brunke **F** antenna of *B.nigropolitoides* Brunke. Scale bars: 1 mm (**A, B**); 0.5 mm (**D–F**).

Kraatz (1858) described *B.erythrurus* based on an unknown number of specimens from “Nova Grenada” [= Panama + Colombia]. Brunke and Solodovnikov (2014) were able to examine one female syntype from the Kraatz collection (SDEI) and redescribed the species based on this and several other, more recently collected single female specimens from medium elevations (1300–1350 m) in southern Puntarenas, Costa Rica and adjacent Chiriquí, Panama. However, the non-syntype females differed from the syntype in coloration, with the former group possessing a bluish forebody and brassy-green elytra (Fig. 1A), and the latter a brassy-green forebody and bluish elytra (Fig. 1B). The legs of the syntype also appeared darker and less clearly bicolored than the non-syntypes and most species of the genus (Fig. 1B), though this was thought to be due to age or discoloration from a killing agent (Brunke and Solodovnikov 2014). The above specimen from IRSNB (Fig. 1C), also collected from “Nova Grenada”, corresponds closely with the syntype in coloration, including the faintly but clearly bicolored legs with a paler area in the basal third of the femur. This raises the possibility that *B.erythrurus* was originally described from present-day Colombia rather than Panama and may not be conspecific with the specimens previously examined from Central America. This hypothesis is further supported by an image taken by D. Hoyos Velasquez of a live specimen from La Tebaida, Quindío, Colombia (mid-elevation, northern Andes), matching the coloration of specimens from “Nova Grenada” and posted on various social media websites (e.g., https://www.facebook.com/groups/491915904153407/posts/2464208683590776). This image represents the first definitive evidence of the Buphthalmus group in South America. A more accurate species concept for *B.erythrurus* is badly needed but will not be possible without the study of male specimens from both Central and South America.

Although the specimen from IRSNB is quite old and collected from “Nova Grenada”, it is not considered to be a syntype of *Cyrtothoraxerythrurus* as the labels are different from that of the syntype deposited in the Kraatz collection (Brunke and Solodovnikov 2014).

#### ﻿ Bolitogyrus sallei

Taxon classificationAnimaliaColeopteraStaphylinidae

(Kraatz, 1858)

7480FA1B-FB04-5045-A27D-6FDB99909172

Cyrtothorax sallei Kraatz, 1858: 367
Bolitogyrus sallei (Kraatz): Brunke and Solodovnikov 2014 (resurrection, redescription)

##### Non-type material.

**Mexico: Veracruz**: “Jalappa” [= Xalapa] (1, NMPC).

##### Comment.

Only two distant localities were previously known for this species: Chapulhuacán (Hidalgo) and Córdoba (Veracruz) (Brunke and Solodovnikov 2014).

#### ﻿ Bolitogyrus salvini

Taxon classificationAnimaliaColeopteraStaphylinidae

(Sharp, 1884)

7204D61B-EDE5-5876-8E91-F9B1865F2F21

Cyrtothorax salvini Sharp, 1884: 341
Bolitogyrus salvini (Sharp): Brunke and Solodovnikov 2014 (redescription; three morphotypes)

##### Non-type material.

**Guatemala: Suchitepéquez**: Volcán Atitlán, Ref. El Quetzal, 1670 m, 14.55067, –91.19235, 3–6.VI.2015, Z.H. Falin & F. Carrillo, ex. FIT [flight intercept trap], cloud forest (1 male, SEMC).

##### Comment.

The above specimen perfectly corresponds to *B.salvini* morphotype I of Brunke and Solodovnikov (2014): forebody with greenish metallic reflections, disc of head without impunctate area, frontal impression weak, basal segments of abdomen reddish, apex of abdomen pale, parameral arms with dense peg setae. The only other known specimens of morphotype I are the male and female types from El Zapote, Guatemala, also in the Sierra Madre mountain range. The close morphological consistency of the non-type male with the type series suggests that the concept of *B.salvini* should be restricted to morphotype I alone. However, formal changes, including the possible description of new species corresponding to morphotypes II and III, should wait until the unknown male of morphotype II is studied.

### ﻿Bullatus lineage

**Strigifrons group**

#### ﻿Revised key to the Strigifrons group

**Table:** 

1	Base of head with a pair of large, glossy protuberances, creating expansive impunctate areas (fig. 6A in Brunke and Solodovnikov 2014); Guatemala	***B.silex* Brunke**
–	Base of head with small, well separated protuberances that may or may not be entirely obscured by sculpture; Mexico	**2**
2	Head with small, shining, protuberances generally lacking sculpture; pronotum laterally without strigose sculpture forming longitudinal channels (Fig. 1D); abdominal tergite VI without median impunctate area	***B.viridescens* Brunke**
–	Head with small protuberances, almost entirely obscured by sculpture; pronotum laterally with strigose sculpture forming longitudinal channels (Fig. 1E); abdominal tergite VI with median impunctate area	**3**
3	Elytra with extensive strigose sculpture, not limited to lateral patch; abdominal tergite VII with median impunctate area; paramere longer than median lobe and with peg setae arranged in a simple, well-aligned marginal row at each side (Fig. 2E); apex of median lobe in ventral view narrower and more strongly converging to apex (Fig. 2C)	***B.strigifrons* (Wendeler), sensu nov.**
–	Elytra with strigose sculpture limited to lateral patch; abdominal tergite VII without median impunctate area; paramere shorter than median lobe and with peg setae arranged in loosely organized marginal row, often with doubled punctures (Fig. 2F); apex of median lobe in ventral view broader and more weakly converging to apex (Fig. 2D)	***B.pseudostrigifrons* Brunke, sp. nov.**

#### ﻿ Bolitogyrus pseudostrigifrons

Taxon classificationAnimaliaColeopteraStaphylinidae

Brunke sp. nov.

422B7A02-9D67-5305-B9FD-54B327AF4938

http://zoobank.org/CC047B84-4978-4F62-8C1F-ACD9DBE8A307

Figs 1E , 2B, D, F

Bolitogyrus strigifrons (Wendeler): Brunke and Solodovnikov, 2014 (misidentification, in part)

##### Type locality.

Tlanchinol, 43 km SW Huejutla de Reyes, Hidalgo, Mexico.

##### Type material.

***Holotype*** (male, CNC): Mex: Hdgo., Tlanchinol, 43 km SW Huejutla, 14.VI.–4.VIII.1983, S.&J. Peck, 1500 m, cloud forest FIT [typed label] / *Bolitogyruspseudostrigifrons* Brunke, des. Brunke, 2021 [red label].

***Paratypes*** (4, CNC; 1 UAEH): same data as holotype (2 males, 2 females, CNC); **Hidalgo**: Zacualtipán, Camino a Sto. Domingo [trail to Santo Domingo], 20°38'00.7"N, 98°34'00.5"W, 1830 m, Bosque mixto? o mesofilo? [=mixed? or cloud forest?] pert. [=disturbed], en troncos podridos [in rotten logs], 16.VIII.2003, J. Asiain y J. Márquez (1 male, UAEH).

##### Etymology.

The species epithet refers to the similarity to its sister species, *B.strigifrons* (Wendeler).

##### Diagnosis.

**Figure 2.**
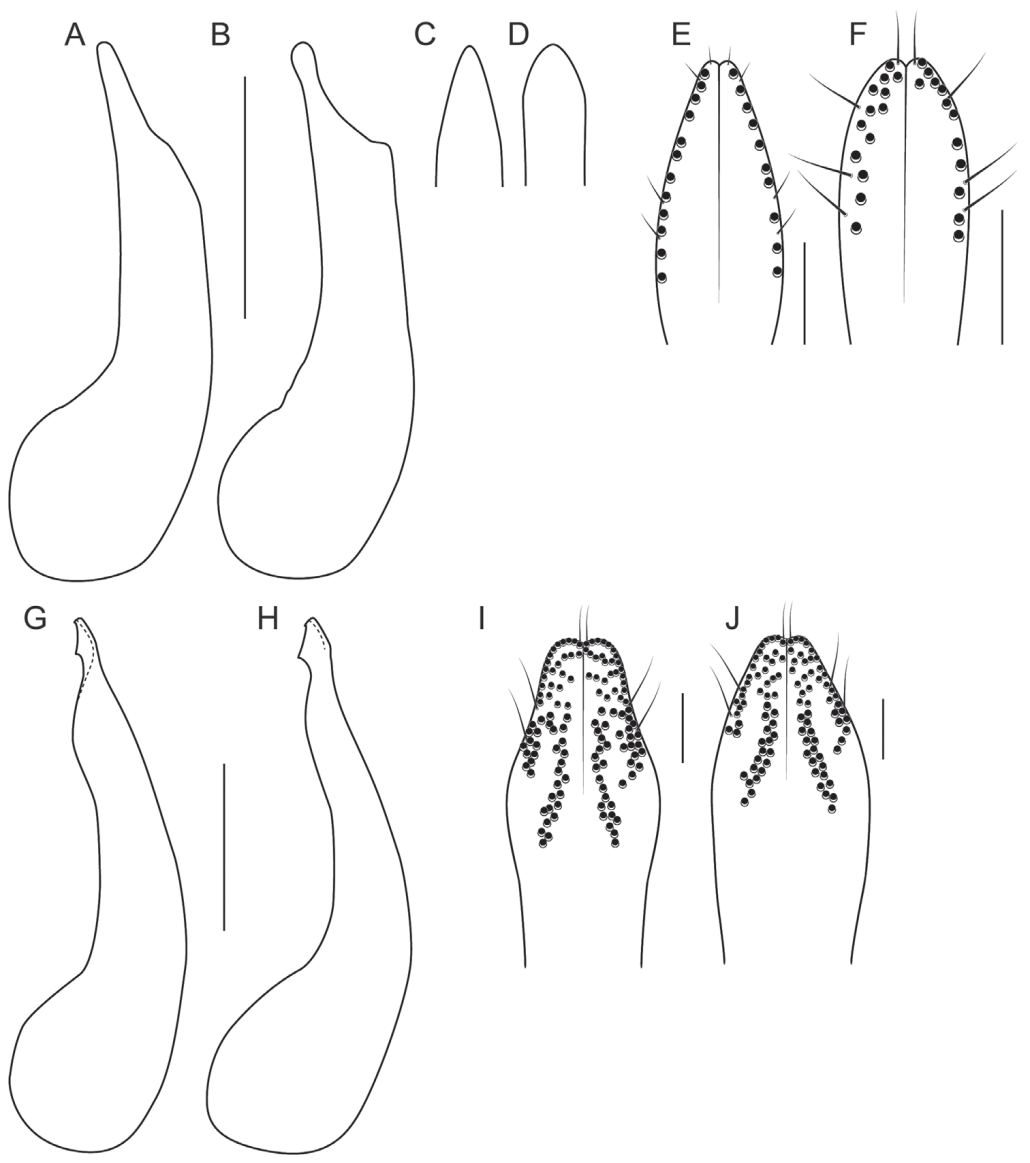
Male genitalia **A, C, E***Bolitogyrusstrigifrons* (Wendeler) **B, D, F***B.pseudostrigifrons* Brunke **G, I***B.nigropolitus* Smetana **H, J***B.nigropolitoides***A, B, G, H** median lobe in lateral and **C, D** ventral view. **E, F, I, J** inner face of paramere, apex. Scale bars: 0.5 mm (**A–D, G, H**); 0.1 mm (**E, F, I, J**).

Within the Strigifrons group (for diagnosis, see Brunke and Solodovnikov (2014)): strigulose sculpture of elytra present but restricted to small lateral patch; posterior protuberances of head not creating expansive impunctate areas; abdominal tergite VI (but not VII) with disc impunctate medially. *Bolitogyruspseudostrigifrons* is most similar to *B.strigifrons* but can be easily recognized externally by the lack of an impunctate medial area on tergite VII and the strigulose sculpture on the elytra limited to a lateral patch. The paramere is also markedly different (Fig. 2F), with the overall shape expanded subapically and with disorganized, marginal rows of peg setae that are often doubled; it is also shorter than the median lobe, while it is longer in *B.strigifrons*. Additionally, the apex of the median lobe in ventral view is broader and less strongly convergent (Fig. 2D).

##### Description.

Measurements ♂ (*n* = 4): HW/HL 1.54–1.60; PW/PL 1.42–1.56; EW/EL 1.20–1.35; PW/HW 1.11–1.13; ESut/PL 0.82–0.90; forebody length 3.26–3.58 mm.

Measurements ♀ (*n* = 2): HW/HL 1.50–1.55; PW/PL 1.46–1.50; EW/EL 1.32–1.44; PW/HW 1.10; ESut/PL 0.82–0.88; forebody length 3.26–3.37 mm.

As in the description of *B.strigifrons* given by Brunke and Solodovnikov (2014) except: head distinctly more transverse (*B.strigifrons*, HW/HL = 1.38); elytra with strigose microsculpture confined to lateral patch; tergite VII without clear impunctate medial area; aedeagus with paramere distinctly shorter than median lobe; median lobe in ventral view broader, less strongly convergent to apex (Fig. 2D); median lobe in lateral view with apex slightly swollen, knob-like (Fig. 2B); paramere spoon-shaped, apical part broadest subapically, with marginal row of peg setae disorganized and with several setae in row doubled (Fig. 2F), parameral setae thicker and longer.

##### Distribution.

This species is known from two rather close localities in Hidalgo, Mexico.

##### Bionomics.

Specimens were collected in cloud forests (1500–1830 m), using an FIT and from a rotten log.

##### Comments.

In Brunke and Solodovnikov (2014), the only non-type specimen of *B.strigifrons* available (male from Hidalgo, listed as a paratype above) had the tip of the paramere missing. Although there were some external differences with the holotype that were noted, a conservative approach was taken, and they were treated as conspecific. A newly available series of specimens from Hidalgo (CNC), in good condition, revealed that two species are involved that differ both externally and in male genitalia. This is the species figured in Brunke and Solodovnikov (2014) as *B.strigifrons* (fig. 6F, 13E).

#### ﻿ Bolitogyrus strigifrons

Taxon classificationAnimaliaColeopteraStaphylinidae

(Wendeler, 1928), sensu nov.

FB70E3DB-458F-5871-ADA3-9465E146A626

Fig. 2A, C, E

Cyrtothorax strigifrons Wendeler, 1928: 34
Bolitogyrus strigifrons (Wendeler): Brunke and Solodovnikov 2014 (redescription; misidentification of B.pseudostrigifrons, in part)

##### Type material.

Holotype, examined for Brunke and Solodovnikov (2014), images used for comparison.

##### Diagnosis.

Within the Strigifrons group (for diagnosis, see Brunke and Solodovnikov (2014)): strigulose sculpture of elytra present and more expansive, not restricted to small lateral patch; posterior protuberances of head not creating expansive impunctate areas; abdominal tergites VI–VII with disc impunctate medially. *Bolitogyrusstrigifrons* is most similar to *B.pseudostrigifrons* but can be easily recognized externally by the impunctate medial area on tergite VII and the more expansive strigulose sculpture on the elytra. The paramere is also markedly different (Fig. 2E), with the overall shape evenly narrowed to the apex and with simple, organized marginal rows of peg setae; it is also longer than the median lobe. Additionally, the median lobe in ventral view is more strongly convergent to a narrower apex (Fig. 2C)

##### Distribution.

In its revised sense, *B.strigifrons* is known only from the holotype male, collected from an imprecise locality in Veracruz state, Mexico (“Jalapa”).

##### Comments.

At the moment, sister species *B.pseudostrigifrons* (Hidalgo) and *B.strigifrons* (Veracruz) appear to be broadly allopatric but more collecting is needed in Veracruz and Puebla states at medium elevations to verify this.

#### ﻿ Bolitogyrus viridescens

Taxon classificationAnimaliaColeopteraStaphylinidae

Brunke, 2014

94B89A6F-C356-523A-BAA4-3AAF95A4464F

Bolitogyrus viridescens Brunke in Brunke and Solodovnikov 2014: 73

##### Non-type material.

**Mexico: Veracruz**: “Jalapa”, Georg Heine (1 female, NMPC); Aguita Fria, 1.3 km SWW of Rancho Viejo (W of Xalapa), 19°31.3'N, 96°59.5'W, 1510 m, 9.IX.2016, Alvarado, Arriaga, Fikáček and Seidel lgt., sifting of thin layer of leaf litter, sparse riverside forest with emergent, large *Platanus* and dense understory (1 female, NHMD).

##### Comment.

*Bolitogyrusviridescens* was previously known only from a single imprecise locality: ~9 km E Teziutlán. Although this locality was listed as from Puebla state on the labels of the type series (Brunke and Solodovnikov 2014), 9 km E of Teziutlán must be just over the border in neighboring Veracruz state. Based on the detailed record from Aguita Fria, *B.viridescens* is probably sympatric with *B.strigifrons*, and both are recorded from the general area around the city of Xalapa.

### ﻿Neotropical species incertae sedis

#### ﻿ Bolitogyrus newtoni

Taxon classificationAnimaliaColeopteraStaphylinidae

Brunke, 2014

D5683D42-26A9-54CB-9B1D-62269E94760E

Bolitogyrus newtoni Brunke in Brunke and Solodovnikov 2014: 78

##### Non-type material.

**Guatemala: Suchitepéqez**: Volcan Atitlán, Ref. El Quetzal, 1670 m, 14.55067, −91.19235, 6–10.VI.2015, Z.H. Falin & F. Carrillo, ex. FIT, cloud forest (1, SEMC); same except 10–13.VI.2015 (2, SEMC); same except 13–16.VI.2015 (1, SEMC).

##### Comments.

The above material, more than doubling the number of known specimens, was collected by FIT very close to the locality of one paratype and indicate that this species readily disperses through flight.

### ﻿Oriental species

**Electus group**

#### ﻿Revised key to the Electus group

Upon study of one paratype of *B.cyanipennis* (cSmet), it was discovered that this species can also have a darker abdomen, with only the apical margins of the tergites paler. The key below is emended below to accommodate for this color dimorphism.

**Table:** 

1	Pronotum dark reddish brown to bright orange, contrasting with dark head; elytra bright metallic green to blue; abdomen bicolored red and black, or at least with tergites distinctly paler apically	**2**
–	Pronotum dark, concolorous with head; elytra with only faint metallic reflection; abdomen entirely dark, elytra dark	**3**
2	Paramere with constricted stem, exposing median lobe in parameral view; apex of median lobe obtuse in parameral view; northeastern Sichuan, northern Chongqing and southern Shaanxi, China	***B.kitawakii* Smetana & Zheng**
–	Paramere vaguely constricted, not exposing median lobe in parameral view; apex of median lobe acute in parameral view; north-central Sichuan, China	***B.cyanipennis* (Zheng)**
3	Head with deeply impressed punctures, many punctures confluent, forming rows; Hubei and Guizhou, China	**4**
–	Head with regular, non-impressed punctures, most punctures clearly separated, Sichuan and Yunnan, China, Laos and Vietnam	**5**
4	Paramere with peg setae medially, on projected ridge; peg setae with median group extended clearly basad of marginal group; median lobe in lateral view without subapical teeth; Hubei, China	***B.metallicus* Cai et al.**
–	Paramere without projected ridge; peg setae with median group extended to no more than just behind level of marginal group; median lobe in lateral view with small subapical teeth; Guizhou, China	***B.nigerrimus* Yuan et al.**
5	Hind tibia in lateral view with at least distal half distinctly paler than darkened portion of femur; paramere with median rows of peg setae extended far basad of marginal rows (Fig. 2I, J); median lobe in lateral view subparallel in middle portion (Fig. 2G, H)	**6**
–	Hind tibia in lateral view entirely dark, as dark as darkened portion of femur; paramere with median rows of peg setae, if present, extended only just basad of marginal rows; median lobe in lateral view gradually widening basad from subapex	**7**
6	Antennomere 5 elongate, 6 very weakly transverse; paramere shorter than median lobe, apex of median lobe visible in parameral view; paramere with wide subapical part angulate and then narrowed to broader, more truncate apex (Fig. 2I); median lobe in lateral view with large subapical expansion (Fig. 2G); Sichuan, China	***B.nigropolitus* Smetana**
–	Antennomere 5 subquadrate, 6 distinctly transverse (Fig. 1F); paramere slightly longer than median lobe, apex of median lobe not visible in parameral view; paramere with wide subapical part broadly rounded to narrower apex (Fig. 2J); median lobe in lateral view with only slight subapical expansion (Fig. 2H); northern Vietnam	***B.nigropolitoides* Brunke, sp. nov.**
7	Antennomeres 7–10 relatively elongate: 6 quadrate and 7 weakly transverse; paramere with attenuate apex	**8**
–	Antennomeres 7–10 relatively transverse: 6 weakly, and 7 distinctly transverse; paramere with evenly converging sides	**9**
8	Apex of median lobe in lateral view forming a more elongate triangle; paramere in lateral view with broad lateral projection; Central Yunnan, China, east of the Salween River	***B.electus* Smetana & Zheng**
–	Apex of median lobe in lateral view forming a shorter triangle; paramere in lateral view with sharp lateral projection; Western Yunnan, China, west of the Salween River	***B.uncus* Cai et al.**
9	Peg setae absent from broad oval shaped area along middle of paramere; median lobe in lateral view without expansion basad of subapical tooth; Western Yunnan, China, west of the Salween River, possibly adjacent Myanmar	***B.huanghaoi* Hu et al.**
–	Peg setae absent from only narrow strip along middle of paramere; median lobe in lateral view with distinct expansion basad of subapical tooth; southeast Yunnan, China, and northern Laos and Vietnam (possibly northern Thailand)	***B.confusus* Brunke**

#### ﻿ Bolitogyrus confusus

Taxon classificationAnimaliaColeopteraStaphylinidae

Brunke, 2017

BE8851CC-E733-593B-84D2-DC48A67C3A6B

Bolitogyrus confusus Brunke, 2017: 18
Bolitogyrus electus Smetana & Zheng, 2000: Hu et al. 2011 (misidentification)

##### Non-type material.

**Vietnam: Lai Châu**: Hoàng Liên Nat. Pk., Tram Ton Pass

22.348, 103.775, 1948 m, subtropical forest, fungusy wood, beating, 23.VI.2017, R. Schuh (1 male, 1 female, CNC).

##### Comment.

Newly recorded from Vietnam and known elsewhere from southern Yunnan, China and northern Laos. The above specimens fill in a distribution gap between the southern Chinese and Laos localities. *Bolitogyrusconfusus* may also occur in northern Thailand. The specimens were collected by beating wooden trail posts that had weathered, cracked, and become fungusy in the constant humidity.

#### ﻿ Bolitogyrus huanghaoi

Taxon classificationAnimaliaColeopteraStaphylinidae

Hu et al., 2011

1F8201D7-4C85-53DA-9301-B1289CE7A77D

Bolitogyrus huanghaoi Hu et al., 2011: 60
Bolitogyrus huanghaoi Hu et al.: Brunke 2017 (redescription)

##### Non-type material.

**China: Yunnan**: 1.8 km W Zizi vill., 2.VII.2016, 25°44.7'N, 98°33.6'E, 2005 m, from large dead tree stumps, J. Hajek and J. Ruzicka (1 female, NMPC).

##### Comments.

Although this specimen is a single female, the transverse antennomeres and its locality west of the Salween River (“Nujiang” in China) allow for a determination to *B.huanghaoi*. The specimen represents only the third record for this species, just west (~13 km) of the type locality. Another single female is known from just over the border in Myanmar (Brunke and Solodovnikov 2014).

#### ﻿ Bolitogyrus uncus

Taxon classificationAnimaliaColeopteraStaphylinidae

Cai et al., 2015

3BA4E565-1142-558E-A30D-87D5531B0F83

Bolitogyrus uncus Cai et al., 2015: 472
Bolitogyrus uncus Cai et al.: Brunke et al. 2017 (redescription)

##### Non-type material.

**China: Yunnan**: Gaoligong mts, 2200–2500 m, 24.57, 98.45, 8–16.V.1995, O. Semela (5, cShi).

##### Comments.

The female paratype of sister species *B.electus*, collected from Gaoligongshan, was considered of doubtful identity by Brunke (2017) based on it being a western outlier from the main distribution of that species. A series of males and females from the same locality indicate that this female paratype is really *B.uncus* and that these two species are probably separated allopatrically by the Salween River valley (“Nujiang” in China).

#### ﻿ Bolitogyrus nigropolitoides

Taxon classificationAnimaliaColeopteraStaphylinidae

Brunke sp. nov.

F3090613-E01A-5617-8B77-CE8B5D2923CB

http://zoobank.org/E2752AD1-20AB-49C7-ADBB-86EEFA76FDCE

Figs 1F , 2H, J

##### Type locality.

Phia Oac National Park, Cao Bang, Vietnam.

##### Type material.

***Holotype*** (male, CNC): Vietnam, Cao Bang, Phia Oac Nat. Park, summit rd., behind upper FIT, 22°36'21.60"N, 105°52'19.20"E, 1600 m, mat. secondary forest, fogging standing dead tree w/ orange fungus, 7–17.V.2019, A. Brunke & H. Schillhammer, CNC1898850 [typed label] / *Bolitogyrusnigropolitoides* Brunke, des. Brunke, 2021 [red label]

##### Etymology.

The species epithet refers to the similar species *B.nigropolitus* from Sichuan, China.

##### Diagnosis.

Within the Electus group (for diagnosis, see Brunke and Solodovnikov 2014): abdomen entirely dark; head without deeply impressed punctures; lateral face of hind tibia bicolored, distal half distinctly paler than darkened part of hind femur; antennomere 5 subquadrate, 6 distinctly transverse (Fig. 1F); paramere slightly longer than median lobe, in ventral view median lobe not visible; paramere with apical part broadly rounded to apex (Fig. 2J); median lobe in lateral view with smaller subapical expansion (Fig. 2H).

##### Description.

Measurements ♂ (*n* = 1): HW/HL 1.30; PW/PL 1.17; EW/EL 1.12; ESut/PL 0.85; PW/HW 1.09; forebody length 4.5 mm.

Very similar to *Bolitogyrusnigropolitus* (Fig. 2G, 2I) except: antennae shorter, antennomere 5 subquadrate, 6 distinctly transverse (Fig. 1F); pronotum slightly less transverse; elytral slightly longer; paramere slightly longer than median lobe, in ventral view median lobe not visible with paramere in situ; paramere with apical part broadly rounded to narrower apex (Fig. 2J); median lobe in lateral view with subapical expansion much smaller (Fig. 2H).

##### Distribution.

This species is known only from Phia Oac National Park in northern Vietnam, though it likely occurs at similar elevations in neighboring Yunnan, China and elsewhere in northern Vietnam east of the Red River.

##### Bionomics.

The holotype was pyrethrin-fogged from a dead standing tree, bearing orange-fungal fruiting bodies.

###### Caesareus group

#### ﻿ Bolitogyrus

Taxon classificationAnimaliaColeopteraStaphylinidae

sp.

79A8C3B8-13D0-5C63-B847-E827D8433C14

##### Non-type material.

**Indonesia: Sumatra**: Aceh, G. Leuser Nat. Pk., Ketambe Res. Sta., 3°41'N, 97°39'E, primary rainforest, malaise trap, 350 m, XII.1989, IIS 89001B, D.C. Darling, 1 female (ROM).

##### Comments.

The above single female specimen is similar to *B.proximus* but could also represent an undescribed species. The genus is newly reported from the island of Sumatra, where it is undoubtedly diverse but extremely poorly sampled.

###### Vulneratus group

#### ﻿ Bolitogyrus flavus

Taxon classificationAnimaliaColeopteraStaphylinidae

Yuan et al., 2007

007FD8A0-C4CD-59BC-92DB-27E83AFF7709

Bolitogyrus flavus Yuan et al., 2007: 148
Bolitogyrus flavus Yuan et al.: Brunke 2017 (redescription)

##### Non-type material.

**Vietnam: Hoa Bihn**: “Tonkin”, de Cooman (1, NMPC).

#### ﻿ Bolitogyrus depressus

Taxon classificationAnimaliaColeopteraStaphylinidae

Cai et al., 2015

1615027B-42D9-5F97-AA24-F32FE8EDCA88

Bolitogyrus depressus Cai et al., 2015: 454
Bolitogyrus depressus Cai et al.: Brunke 2017 (redescription)

##### Non-type material.

**China: Guangdong**: Nanling Nat. Reserve, Dadongshan, 18–21.IV.2013, border of mixed forest, 24°56.0'N, 112°42.9'E, 690 m, J. Hajek and J. Ruzicka (1, NMPC); **Guangxi**: Longsheng Hot Spring, forested river valley, wet rocks, 25°53.6'N, 110°12.4'E, 360 m, 11–14.IV.2013, M. Fikacek, J.Hajek, J. Ruzicka (2, NMPC).

##### Comments.

This species is newly reported from Guangxi, indicating that it is probably very broadly distributed in the low forested hills of southeastern China. The specimens from wet rocks in a forested river valley probably dropped when disturbed from overhanging coarse woody debris.

#### Bolitogyrus tumidus

Taxon classificationAnimaliaColeopteraStaphylinidae

Brunke, 2017

4E8108B0-F743-5729-9E3D-DAE1EBBACD87

Bolitogyrus tumidus Brunke, 2017: 55

##### Non-type material.

**Laos: Hua Phan**: 20°13'9–19"N, 103°59'54–104°0'3"E, 1480–1510 m, Phou Pane Mt. [= Mt. Phu Phan], 22.IV.–14.V.2008, Vit. Kuban (3, NMPC).

#### ﻿ Bolitogyrus himalayicus

Taxon classificationAnimaliaColeopteraStaphylinidae

Brunke, 2017

97406C90-4069-5F88-87F1-25B0ADFA137A

Bolitogyrus himalayicus Brunke, 2017: 66
Bolitogyrus vulneratus Fauvel, 1878: Smetana 1988 (misidentification of B.himalayicus)

##### Non-type material.

**Nepal: Prov. No. 1**: Khandbari, Arun Valley, at Num bridge, 1050 m, 22.IV.1984, Smetana and Löbl (1 male, cSmet).

##### Comments.

*Bolitogyrushimalayicus* is here newly reported from Nepal. The above specimen is substantially larger than the holotype and was collected at a higher elevation (200 m versus 1000 m), but its aedeagal morphology corresponds perfectly. This specimen also represents the only record of the genus from Nepal and was previously reported by Smetana (1988) under the name *B.vulneratus*. The couplets from the key in Brunke (2017) should be modified as follows:

**Table:** 

30	Paramere with subbasal expansion in lateral view; Garo Hills, Meghalaya, India	***B.concavus* Brunke**
–	Paramere without subbasal expansion in lateral view; Khasi Hills, Meghalaya, and Himalaya of Nepal and West Bengal, India	**31**
31	Apex of the median lobe in parameral view with single toothed carina; paramere with peg setae arranged in disorganized marginal row, apex with dense group; Himalaya of Nepal and West Bengal, India	***B.himalayicus* Brunke**
–	Apex of median lobe in parameral view with double-toothed carina; paramere with peg setae in sparse, single marginal row; Khasi Hills, Meghalaya, India	***B.nanus* Brunke**

## Supplementary Material

XML Treatment for Bolitogyrus

XML Treatment for Bolitogyrus costaricensis

XML Treatment for Bolitogyrus erythrurus

XML Treatment for Bolitogyrus sallei

XML Treatment for Bolitogyrus salvini

XML Treatment for Bolitogyrus pseudostrigifrons

XML Treatment for Bolitogyrus strigifrons

XML Treatment for Bolitogyrus viridescens

XML Treatment for Bolitogyrus newtoni

XML Treatment for Bolitogyrus confusus

XML Treatment for Bolitogyrus huanghaoi

XML Treatment for Bolitogyrus uncus

XML Treatment for Bolitogyrus nigropolitoides

XML Treatment for Bolitogyrus

XML Treatment for Bolitogyrus flavus

XML Treatment for Bolitogyrus depressus

XML Treatment for Bolitogyrus tumidus

XML Treatment for Bolitogyrus himalayicus
